# HIV-child with disseminated *Talaromyces Marneffei* (*Penicillium marneffei*) infection: a rare severe case report

**DOI:** 10.1186/s12887-021-03100-5

**Published:** 2022-01-07

**Authors:** Wei Hu, Xiao-hong Yu, Wei-qin Wei, Xuan Xiang

**Affiliations:** 1 Emergency department, Guiyang Second People’s Hospital, Guiyang, Guizhou Province China; 2 Emergency department, Affiliated Hospital of Guizhou Medical University, Guiyang, Guizhou Province China

**Keywords:** HIV-child, Penicilliosis, Talaromyces marneffei, AIDS, Smear analysis, Rare disease

## Abstract

**Background:**

*Talaromyces Marneffei* (*Penicillium marneffei, T.marneffei*) has been frequently reported in patients with adult acquired immunodeficiency syndrome. Still, cases of children with HIV combined with *T.marneffei* infection are very rare. This report describes the case of a HIV-child who is a girl from China. Her special clinical manifestations and laboratory diagnosis results can provide clinicians with the basis for diagnosis and treatment of *T.marneffei* related rare diseases.

**Case presntation:**

We reported a single case of 7-year-old Chinese female patient who presented with fever, abdominal pain, multiple lymphadenopathy, hepatosplenomegaly, left lower extremity ecchymosis, and bloody stool. The patient received anti-inflammatory therapy; however, her symptoms did not improve. Consequently, she was diagnosed with *T.marneffei* and HIV infection; it was also confirmed that her mother did not undergo HIV blocking therapy during pregnancy. Yet, the child’s family refused all treatment, after which the child was discharged from the hospital. The patient died a few days later.

**Conclusion:**

This case suggested that children with AIDS suffering from fever, lymphadenopathy and coagulation dysfunction, penicilliosis should be suspected. Clinicians should diagnose the disease early through laboratory and imaging results, which can help reduce the mortality, prolong the survival time and improve the quality of life of children.

## Background

*Talaromyces marneffei* (*Penicillium marneffei, T.marneffei*) is a dimorphic fungus often found in HIV and immunocompromised patients [1, 2]. Fungi thrive in a humid climate and are closely associated with bamboo rats [3]. They are geographically confined to the Southern Ocean, especially Southern China [4]. In immunocompromised patients, *T.marneffei* can quickly spread and cause systemic disseminated infection. If left untreated, it can be fatal. Despite most of patients having received antifungal treatment, its current global mortality rate is 27.7% [5, 6]. Mother to child transmission of AIDS in children is a rare disease characterized by acute onset and short incubation period. Herein, we reported a severe case of a 7-year-old girl with AIDS from Guizhou Province, China, who was infected with *T.marneffei*, including her clinical and imaging findings.

## Case presentation

A 7-year and 8-month-old child with untreated HIV that progressed to AIDS living in an epidemic area of southern China consulted the Guiyang maternal and child health hospital. She had a fever with chills and paroxysmal abdominal pain. The pain was mainly located under the xiphoid process. After 4 days of treatment, the patient’s condition did not significantly improve, fever symptoms were still present and she also developed abdominal pain.

On the fifth day, the child was transferred to Intensive care unit (ICU). The patient and her family members were asked about their medical history in detail. The patient had no direct contact with specific plants recently, including rotten sugarcane or bamboo rat and other animals. Physical examination of the patient showed ecchymosis in the left lower limb. Clusters of 4 mm to 7 mm-sized lymph nodes could be palpable under the jaw, neck, armpit, and groin. The texture was firm, the activity was moderate, and the tenderness was obvious. The respiratory sounds of the left lower lung were lower than those of the right lung. Abdominal distention, abdominal circumference of 51.8 cm, abdominal tenderness, and abdominal muscle tension were observed. Under palpation, the liver was about 7.0 cm below the ribs and 8.5 cm below the xiphoid process. The spleen was about 6 cm below the coastal area. There was tenderness in both thighs, and depressed edema in the left lower limb. The highest temperature was 38.5 °C. The patient was then prescribed oral antipyretic drugs and received physical cooling treatment, after which the body temperature returned to normal levels. At the same time, the child was given cefoperazone sulbactam (Main antibacterial spectrum: influenza bacillus, gas bacillus, Morganella, bacili, *Escherichia coli*, flourodi citrate bacillus, *Enterobacter cloacae*, acinetobacter, pneumonia bacillus, etc) anti-infection and vitamin K1 treatment.

**Fig. 1:**
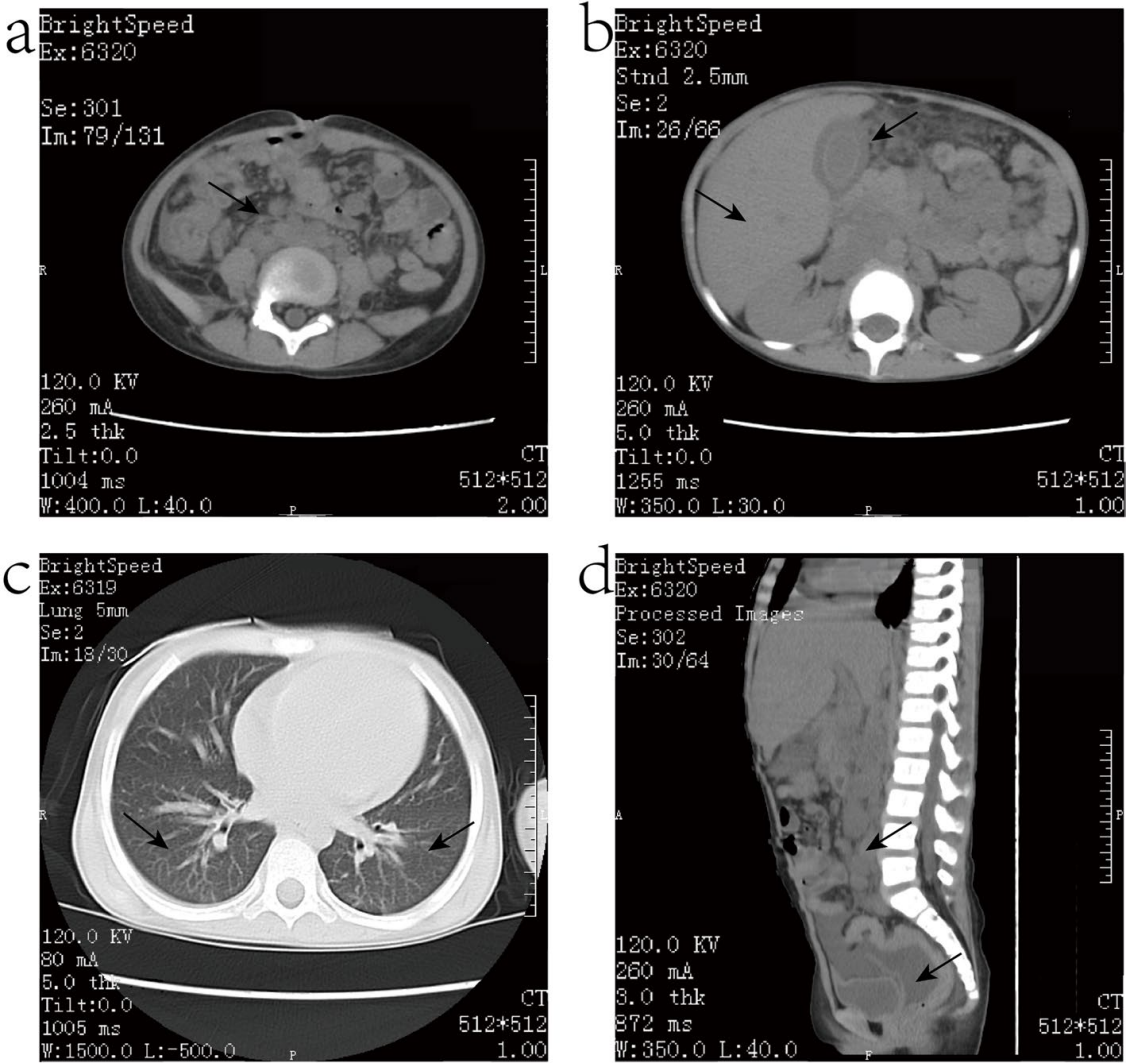
The chest CT manifestations of patient show: 1) multiple enlarged lymph nodes in the abdomen, pelvic cavity, bilateral inguinal region, and axillary region, partially fused into a piece (**a**); 2) diffuse peritonitis, hepatomegaly, splenomegaly, unclear pancreatic head, cholecystitis with effusion in the gallbladder fossa (**b**); 3) lower lobe infection of both lungs (**c**); 4) bilateral pleural, abdominal and pelvic effusion (**d**)

**Fig. 2:**
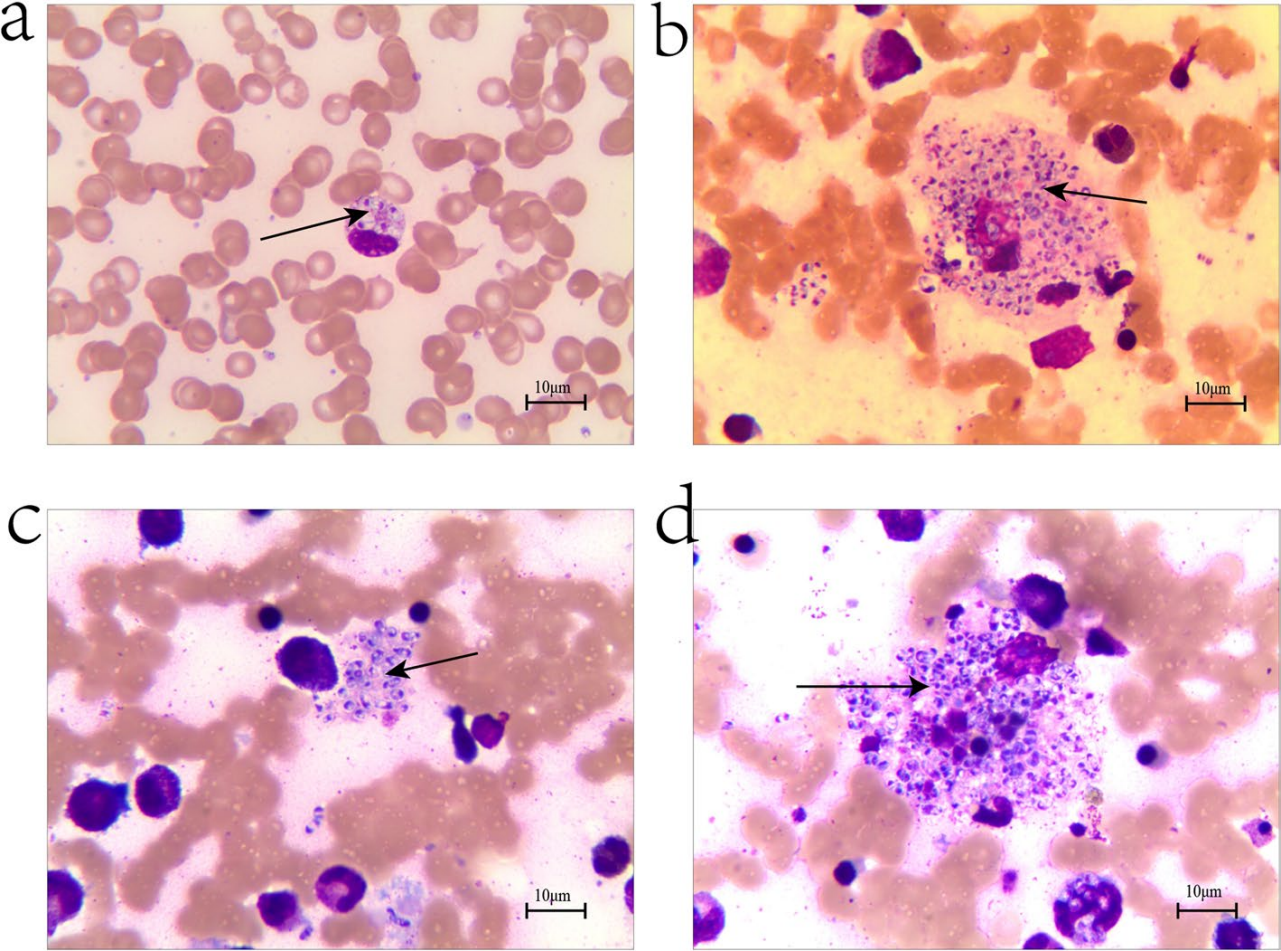
Microscopic appearances of patient show: *T.marneffei* was found in peripheral blood smear (**a**, **b**) and bone marrow smear (**c**, **d**)

The auxiliary examination results showed that the blood cells and Immune cells were decreased: white blood cell count was 3.16 × 10^9^ / L [reference value (4.50–13.50) × 10^9^ / L]. red blood cell count was 3.07 × 10^12^ / L [reference value (4.00–4.50) × 10^12^ /L], hemoglobin was 97 g/L [reference value (110.00–160.00) g/L], platelet count was 22.00 × 10^9^ /L [reference value (125.00–350.00) × 10^9^ /L], CD4/CD8 was 0.07 [reference value (0.98–1.94)], CD3+ was 33.8% [reference value (55.00–78.00) %], CD3+/CD4 was 1.97% [reference value (27.00–53.00) %], CD4 + # was 31.06 cells/μL [reference value (300.00–2000.00) cells/μL]. Cytokines were generally increased: IL-6198.89 pg/mL [reference value (0.00–5.40) pg/mL], IL-10 58.64 pg/mL [reference value (0.00–12.90) pg/mL], IFN- γ 419.23 pg/mL [reference value (0.00–23.10) pg/mL]. Liver function and blood glucose index were abnormal: AST 358.00 U/L [reference value (13.00–35.00) U/L], γ-GGT 110.00 U/L [reference value (7.00–45.00) U/L], LDH 1831.00 U/L [reference value (120.00–250.00) U/L], TBA 89.80 μmol/L [reference value (0.00–10.00) μmol/L], GLU 2.49 mmol/L [reference value (3.88–6.11) mmol/L]. Coagulation function was impaired: PT 18.40 s [reference value (9.80–12.10) s], INR 1.59 [reference value (0.750–1.250)], APTT 51.50 s [reference value (22.70–31.80) s], Fbg 0.47 g/L [reference value (2.00–4.00) g/L], TT 26.80 s [reference value (14.00–21.00) s], FDP 67.09 μg/mL [reference value (0.00–5.00) μg/mL], D-Dimer 36.52 mg/L [reference value (0.00–0.55) mg/L]. Chest and whole abdomen CT showed: 1) multiple enlarged lymph nodes in the abdomen, pelvic cavity, bilateral inguinal region, and axillary region, partially fused into a piece (Fig. 1a); 2) diffuse peritonitis, hepatomegaly, splenomegaly, unclear pancreatic head, cholecystitis with effusion in the gallbladder fossa (Fig. 1b); 3) lower lobe infection of both lungs (Fig. 1c); 4) bilateral pleural, abdominal and pelvic effusion (Fig. 1d). Combined with the above examination results, the patient continued anti-infective treatment of cefoperazone sulbactam, and additionally received creatine phosphate to nourish the myocardium, reduced glutathione to protect the liver, oral ursodeoxycholic acid to treat cholestasis, albumin infusion to correct hypoproteinemia, infusion fresh frozen plasma 200 ml, and platelet 1 treatment volume to improve coagulation function. Eight bone marrow smears and 10 ml bone marrow were collected for examination.

On the same day, clinical laboratory reports confirmed that HIV screening was positive (ELISA). The results were subsequently confirmed by the Centers for Disease Control and Prevention (Western blotting). *T.marneffei* was found in peripheral blood smear (Fig. 2a, b) and bone marrow smear (Fig. 2c, d) by microscope (OLYMPUS CX41, Objective: × 100) and cell graphic analysis system (Jiangsu Jeda, Resolution: 300PPI). After detailed medical history inquiry, it has been discovered that the mother of the child was diagnosed with HIV infection at the time of birth; she did not receive anti-retroviral therapy during pregnancy, and the child was not diagnosed and treated after birth.

After the above treatment, the patient’s abdominal pain and fever symptoms did not improve, and shortness of breath occurred. In addition, the child developed a bloody stool with a frequency of about 7–8 times. Clinical laboratory report: *T.marneffei* was detected in blood culture. On the same day, the child’s family refused all treatment and signed the consent to withdraw treatment, after which the child was discharged. Follow-up showed he patient died 3 days after discharge.

## Discussion and conclusions

Penicilliosis is a deep fungal infectious disease caused by *T.marneffei* infection [7, 8]. It is the second most common invasive deep mycosis of AIDS patients in southern China and Southeast Asia, and the third infectious disease among AIDS patients in South Asian countries, following tuberculosis and cryptococcosis [9]. *T.marneffei* can cause localized mycosis, mainly involving the skin; it can also cause disseminated infection in bone marrow, lung, liver, spleen, and lymph nodes [10]. At present, the transmission route is still unclear. Some studies implicate exposure to soil of epidemic foci in the rainy season and bamboo rat feces are the primary sources of infections in humans [11, 12].

*T.marneffei* has been frequently reported in immunocompromised patients, though very rarely in children with AIDS. Besides, similar cases have never been reported in Guizhou Province, China. Due to the complex and diverse clinical manifestations and lack of specificity, *T.marneffei* can be easily misdiagnosed, affecting the prognosis of patients.

Because of the acute onset and short incubation period of AIDS, CD4+ T cells in children are more vulnerable to damage, which makes the immature immune system of children more susceptible to opportunistic infection. *T.marneffei* mainly invades the reticuloendothelial system of mononuclear macrophages [13]. For patients infected with *T.marneffei*, macrophages phagocytize the infected fungi and reproduce in large quantities. The macrophages containing fungi may easily cause systemic disseminated infection through lymphatic and blood circulation, reaching lymph nodes, liver, spleen, lung and bone marrow, and skin [14]. The main clinical manifestations of these patients are fever, anemia, respiratory symptoms, weight loss, hepatosplenomegaly, systemic lymphadenopathy, and skin damage [10]. Chest imaging can be characterized by diffuse nodules, ground-glass opacity, pleural effusion, multiple mediastinal lymph node enlargements, and similar, which are easily confused with pulmonary tuberculosis, pulmonary cryptococcosis, aspergillosis, and other chronic infectious diseases and malignant pulmonary tumors. Abdominal imaging findings are complex and changeable. Even the same patient may have a variety of imaging manifestations, including abdominal and retroperitoneal lymph node enlargement, hepatosplenomegaly, and ascites.

In this case, a 7-year-old child presented with recurrent fever and abdominal pain. The course of the disease was very short. After treatment, the symptoms were not relieved, and the symptoms of dyspnea and gastrointestinal bleeding appeared in the later stage of the disease. Physical examination showed ecchymosis in the left lower extremity. There were many lymph nodes in the whole body with obvious tenderness. The respiratory sounds of the left lower lung were lower than those of the right lung. In addition, abdominal tenderness, abdominal muscle tension, and hepatosplenomegaly were observed. There was tenderness in both thighs and depressed edema in the left lower limb. The clinical laboratory examination showed that the three blood cell lines were decreased; liver function and blood glucose were abnormal.

Children with clinical manifestations of fever, hepatosplenomegaly, systemic lymphadenopathy, and abnormal hemogram are more likely to be diagnosed with hematological diseases. However, for children who grow up in southern China with the onset time during the summer rainy season, the possibility of infection with *T.marneffei* cannot be ruled out. The children’s medical history and the possibility of HIV infection should be inquired in detail. Moreover, the frequently occurring diseases in the residential area should also be considered. If necessary, the relevant examination should be further improved to avoid misdiagnosis.

The blood routine examination of the subject indicated different degrees of reduction, especially thrombocytopenia. The coagulation test showed that the fibrinogen of the child was significantly decreased, while the coagulation function was obviously abnormal, and the symptoms of gastrointestinal bleeding appeared in the later stage of the course of the disease. However, the reports of *T.marneffei* complicated with thrombocytopenia are rare. In the diagnosis and treatment of this disease, it is necessary to consider whether the patients are complicated with coagulation dysfunction, and be alert to the occurrence of gastrointestinal bleeding or other organs of hemorrhagic diseases that endanger the lives of patients.

Childhood AIDS patients with *T.marneffei* are not typical in clinical practice. After children are infected with *T.marneffei*, the disease develops very rapidly, and the mortality rate significantly increases [15]. Blood bacterial culture is the gold standard for the diagnosis of *T.marneffei* in a microbiological test [16]. However, although this detection method is accurate and has a high detection rate, it requires time to be completed. On the other hand, bone marrow smear tests may be used to detect apparent cell structures/infection inside the bone marrow; however, this method is invasive and not easily performed. In this study, eight bone marrow smears and 10 ml bone marrow were collected for examination. *T.marneffei* was detected in both peripheral blood smear and bone marrow smear. However, bone marrow puncture is an invasive technique, and the more critical the patient is, the more difficult the operation will be. The microscopic examination of the peripheral blood smear is undoubtedly the most economical and rapid test method, which is used as a reference for bone marrow smear test results. Still, the detection rate of the peripheral blood smear is lower than that of the three inspection methods. Therefore, the combination of a peripheral blood smear, bone marrow smear, and bacterial blood culture might be the most accurate and efficient detection approach for *T.marneffei*, which in turn may save valuable time for clinical treatment of such patients, and reduce the mortality and missed detection rate.

International guidelines generally recommend amphotericin B as the treatment of PSM during the induction period for 2 weeks. When CD4+ T cells > 100/μl, choose amphotericin B for 2 weeks induction therapy and itraconazole 400 mg daily for 10 weeks consolidation treatment; when CD4+ T cells < 100/μl, choose amphotericin B for 2 weeks induction therapy and itraconazole traconazole 400 mg daily for 10 weeks consolidation treatment, after which itraconazole 200 mg daily maintenance treatment until CD4+ T cells> 100/μl; patients with normal immune function do not need maintenance treatment; mild patients can be directly treated with itraconazole or oral treatment with voriconazole [17, 18]. The clinical manifestations of this case were non-specific, early diagnosis and treatment were not possible, and this child had multiple organ dysfunctions when she was transferred to our hospital. In addition, there was no rapid diagnosis method, and targeted medications were not available. The condition of this patient progressed rapidly and she eventually died.

Considerable progress has been made towards eliminating HIV among children, but the global burden of HIV infection in children remains a challenge faced by healthcare workers around the world, especially in developing countries [19]. According to data published by the Joint United Nations Programme on HIV/AIDS (UNAIDS) in November 2020, there were approximately 160,000 new cases of HIV infections in infants and children aged 0–9 years in the world in 2018. The total number of HIV-infected children reached 1.12 million, and 770,000 people died from developing opportunistic infections and other HIV-related disorders, and about 15% of them were under 20 years old [20]. In this case, the child was born in 2011. At that time, pregnant women in remote areas of China did not be required to receive routine pregnancy care, which resulted in a marked increase in perinatal HIV transmission and loss of treatment opportunities. In order to prevent progression, the Maternal and Child Health Department of the General Office of the National Health and Family Planning Commission of China issued the Implementation Plan to prevent Mother-to-child Transmission of HIV, Syphilis and Hepatitis B (2015 edition) on April 9, 2015, aiming to achieve the following goals by the end of 2020: ‘The rate of pregnant women for antenatal testing will be over 95%. The rate of the use of antiretroviral agents by pregnant women and their children will be more than 90%.’ According to a report issued by the National Health Commission on November 23, 2018, these policies had significantly reduced perinatal transmission in China from 7.1% in 2012 to 4.9% in 2017. Although the rate of mother-to-child transmission of HIV has decreased, the goal of virtual elimination of perinatal transmission still needs more social service supports. The latest guidelines issued by the Chinese Medical Association suggest that the strategy of whole course management of HIV infection needs the participation of multidisciplinary team in prophylaxis, early diagnosis, individualized treatment and humanistic care [21].

In conclusion, children with AIDS and symptoms, such as fever, abdominal pain, lymphadenopathy, and other clinical manifestations, such as penicilliosis, should be suspected of *T.marneffei*, especially if anti-inflammatory treatment showed to be ineffective. In addition, peripheral blood smear, bone marrow smear, blood bacteria culture can help detecting *T.marneffei*. Besides, our patient showed abnormal hemogram combined with coagulation dysfunction, which suggested that doctors need to be aware of the possibility of hemorrhagic diseases, which should be timely prevented so as to avoid life-threatening conditions. Clinicians should diagnose the disease early through laboratory and imaging results, which can help reduce the mortality, prolong the survival time and improve the quality of life of children.

## Data Availability

The datasets used and/or analysed during the current study are available from the corresponding author on reasonable request.

## Abbreviations

ICU: Intensive Care Unit
AIDS: Acquired Immune Deficiency Syndrome
HIV: Human immunodeficiency virus
IL: Interleukin
IFN: Interferon
AST: Aspartate aminotransferase
γ-GGT: Gamma-glutamyltransferase
LDH: Lactate dehydrogenase
GLU: Glucose
PT: Prothrombin time
INR: International normalized ratio
APTT: Activated partial thromboplastin time
Fbg: Fibrinogen
TT: Thrombin time
FDP: Fibrinogen degradation product
MTCT: Mother-to-child transmission

## References

[1] Tsang CC, Lau SKP, Woo PCY (2019). Sixty years from Segretain's description: what have we learned and should learn about the basic mycology of Talaromyces marneffei?. Mycopathologia..

[2] Ustianowski AP, Sieu TP, Day JN (2008). Penicillium marneffei infection in HIV. Curr Opin Infect Dis.

[3] Liu GN, Huang JS, Zhong XN, Zhang JQ, Zou ZX, Yang ML, Deng JM, Bai J, Li MH, Mao CZ, He ZY (2014). Penicillium marneffei infection within an osteolytic lesion in an HIV-negative patient. Int J Infect Dis.

[4] Li HR, Cai SX, Chen YS, Yu ME, Xu NL, Xie BS, Lin M, Hu XL (2016). Comparison of Talaromyces marneffei infection in human immunodeficiency virus-positive and human immunodeficiency virus-negative patients from Fujian, China. Chin Med J.

[5] Chan JF, Lau SK, Yuen KY, Woo PC (2016). Talaromyces (Penicillium) marneffei infection in non-HIV-infected patients. Emerg. Microbes Infect.

[6] Jiang J, Meng S, Huang S, Ruan Y, Lu X, Li JZ, Wu N, Huang J, Xie Z, Liang B, Deng J, Zhou B, Chen X, Ning C, Liao Y, Wei W, Lai J, Ye L, Wu F, Liang H (2019). Effects of Talaromyces marneffei infection on mortality of HIV/AIDS patients in southern China: a retrospective cohort study. Clin Microbiol Infect.

[7] Matos AC, Alves D, Saraiva S, Soares AS, Soriano T, Figueira L, Fraga F, Matos M, Coelho AC (2019). Isolation of Talaromyces marneffei from the skin of an Egyptian mongoose ( Herpestes ichneumon) in Portugal. J Wildl Dis.

[8] Singh A, Ahmed K, Aydin A, Khan MS, Dasgupta P (2016). Fournier's gangrene. A clinical review. Arch Ital Urol Androl.

[9] Kawila R, Chaiwarith R, Supparatpinyo K. Clinical and laboratory characteristics of penicilliosis marneffei among patients with and without HIV infection in northern Thailand: a retrospective study. BMC Infect Dis. 2013:13–464. 10.1186/1471-2334-13-464 PMC3851520 24094273

[10] Vanittanakom N, Cooper CR, Fisher MC, Sirisanthana T (2006). Penicillium marneffei infection and recent advances in the epidemiology and molecular biology aspects. Clin Microbiol Rev.

[11] Dittus C, Sarosiek S (2017). A case of HIV-negative plasmablastic lymphoma of the bone marrow with a unique immunophenotype. Clin Case Rep.

[12] Huang X, He G, Lu S (2015). Role of Rhizomys pruinosus as anatural animal host of Penicillium marneffei in Guangdong, China [J]. Microb Biotechnol.

[13] Pongpom M, Vanittanakom P, Nimmanee P, Cooper CR, Vanittanakom N (2017). Adaptation to macrophage killing by Talaromyces marneffei. Future Sci OA.

[14] Zeng W, Qiu Y, Lu D, Zhang J, Zhong X, Liu G (2015). A retrospective analysis of 7 human immunodeficiency virus-negative infants infected by Penicillium marneffei. Medicine (Baltimore).

[15] Armstrong-James D, Meintjes G, Brown GD (2014). A neglected epidemic: fungal infections in HIV/AIDS. Trends Microbiol.

[16] Le T, Wolbers M, Chi NH, Quang VM, Chinh NT, Lan NP, Lam PS, Kozal MJ, Shikuma CM, Day JN, Farrar J (2011). Epidemiology, seasonality, and predictors of outcome of AIDS-associated Penicillium marneffei infection in Ho Chi Minh City, Vietnam. Clin Infect Dis.

[17] Nelson M, Dockrell D, Edwards S (2011). British HIV Association, and British Infection Association guidelines for the treatment of opportunistic infection in HIV-seropositive individuals 2011[J]. HIV Med.

[18] Le T, Kinh NV, Cuc NTK (2017). A trial of itraconazole or amphotericin B for HIV-associated talaromycosis [J]. N Engl J Med.

[19] Luzuriaga K, Mofenson LM (2016). Challenges in the elimination of pediatric HIV-1 infection [J]. N Engl J Med.

[20] UNICEF. 2020 World AIDS Day Report. Available at: http://www.childrenandaids.org/sites/default/files/2020-12/2020%20World%20AIDS%20Day%20Report.pdf.

[21] AIDS and Hepatitis C Professional Group, Society of Infectious Diseases, Chinese Medical Association (2021). Chinese guidelines for diagnosis and treatment of HIV/AIDS (2021 edition) [J]. Zhonghua Nei Ke Za Zhi.

